# A rare tongue ulcer in a renal transplant recipient

**DOI:** 10.1016/j.idcr.2020.e00955

**Published:** 2020-09-09

**Authors:** Adham de Kok, Kim L.W. Bunthof, Julian D. Machiels, Ruud G.L. de Sévaux

**Affiliations:** a Department of Nephrology, Radboud University Medical Center, Nijmegen, the Netherlands; b Department of Microbiology & Radboudumc Center for Infectious Diseases, Radboud University Medical Center, Nijmegen, the Netherlands

**Keywords:** Kidney transplant, Tongue, Ulcer, Cryptococcus, Immunosuppression

A 63-year old man with a history of a kidney transplantation in 1997 presented with fever and a painful ulcer on the lateral side of the tongue (Fig. 1). Laboratory results showed no abnormalities except for an elevated C-reactive protein (42 mg/L). Empirical treatment with acyclovir was started and a tongue swab showed a positive result for HSV-1. A biopsy was taken and histopathological examination showed invasion of *Cryptococcus neoformans* (Fig. 2). No signs of malignancy were observed. The cryptococcal antigen test on serum was positive, and negative on cerebrospinal fluid. A CT-scan of the lungs showed extensive ground glass lesions and a lesion of 4.7 cm in the left upper lobe. Bronchoalveolar lavage was positive for *Cryptococcus neoformans* and *Pneumocystis jirovecii.* No malignant cells were seen in the bronchoalveolar lavage fluid. The patient was treated intravenously with a combination of liposomal amphotericin B and flucytosine followed by long term fluconazole and local amphotericin B suspension. High dose co-trimoxazole was initiated to treat the *Pneumocystis jirovecii* pneumonia. Immunosuppression consisting of prednisone and mycophenolate mofetil was temporarily switched to prednisone monotherapy. Over the next 6 weeks the ulcer healed completely. After clinical recovery a follow-up CT scan showed a persistent solid lesion in the lung. Additional analysis revealed a papillary adenocarcinoma, which was treated with curative radiotherapy.

**Fig. 1:**
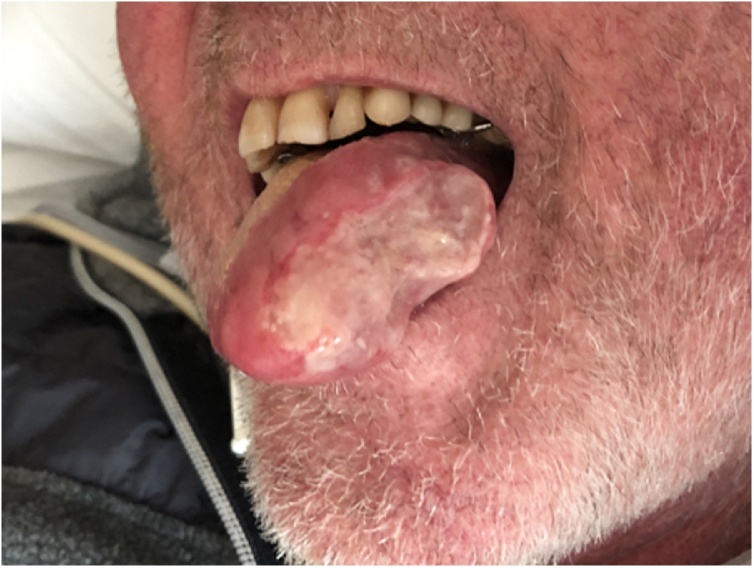
Tongue ulcer.

**Fig. 2:**
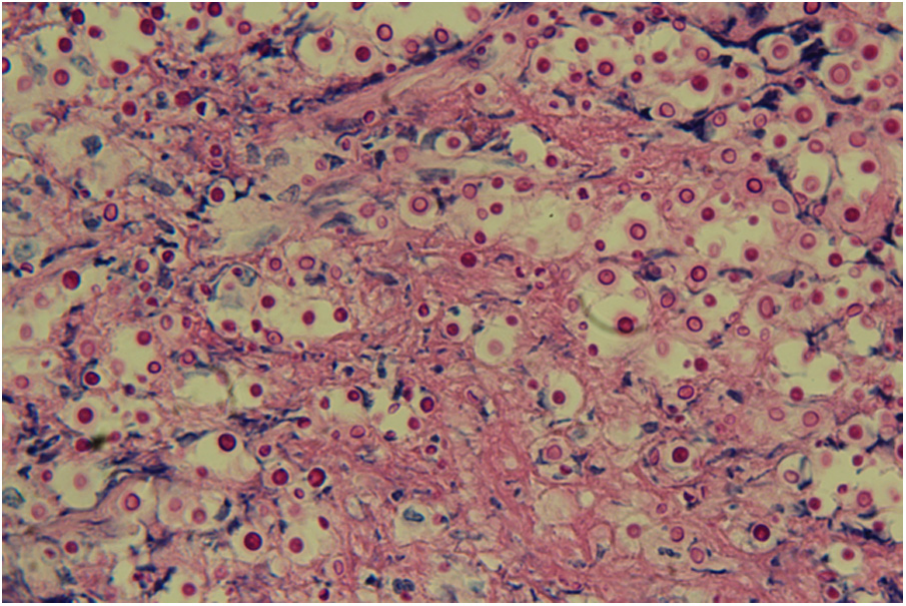
Light microscopy shows invasion of *Cryptococcus neoformans*. Periodic acid–Schiff stain, original magnification x40.

Cryptococcosis is an invasive fungal infection predominantly caused by *Cryptococcus neoformans*. Infection occurs mainly in immunocompromised patients and often leads to meningitis [1]. Other manifestations include pulmonary, dermal and gastrointestinal complaints. Localization of the tongue is a very rare manifestation of this severe infection. Treatment of patients with meningoencephalitis and disseminated disease requires a prolonged course of antifungal therapy [2]. Cryptococcal localizations of the tongue are extremely rare [3]. This report presents a remarkable presentation of disease, twenty-two years after kidney transplantation.

In our opinion, the sudden occurrence of several opportunistic infections many years after transplantation might suggest an underlying disease (malignancy), necessitating an extensive work-up.

## Contributors

AK contributed to patient care and drafting of this manuscript. All Authors were directly involved in clinical management of the patient and revision of the manuscript. All authors approved the final version of this manuscript.

## Transparency document

Transparency document

## Declaration of Competing Interest

The authors report no declarations of interest.
